# Correction: ONC212 is a Novel Mitocan Acting Synergistically with Glycolysis Inhibition in Pancreatic Cancer

**DOI:** 10.1158/1535-7163.MCT-26-0613

**Published:** 2026-08-04

**Authors:** Isacco Ferrarini, Anna Louie, Lanlan Zhou, Wafik S. El-Deiry

In the original version of this article (1), Figs. 1A, D, and 3B contained western blot errors.

Figure 1A and D contained the same Ran bands for the Capan-2 cell line. The Ran bands presented in Fig. 1D were rotated 180 degrees with an altered aspect ratio with respect to the Ran bands present in Fig. 1A. The authors explained this was an error and that the same loading control should have been displayed in Figs. 1A, D, and 2A for visualization reasons. Figure 2A contains the correct presentation of the Ran control and Fig. 1A and D have been corrected. The original image for Ran of the Capan-2 cell line is included within Supplementary Fig. S1 for this notice.

Additionally, the Ran loading control for the BxPC3 cell line in Fig. 3B was a duplicate of the Ran loading control for the Capan-2 cell line in Supplementary Fig. S3. The authors explained that the BxPC3 loading control in Fig. 3B was an error and provided the correct Ran panel. The original image for Ran of the BxPC3 cell line is included within Supplementary Fig. S1 for this notice.

The errors have been corrected in the latest online HTML and PDF versions of the article. The authors regret these errors.

## References

[1] Ferrarini I , Louie A, Zhou L, El-Deiry W. ONC212 is a novel mitocan acting synergistically with glycolysis inhibition in pancreatic cancer. Mol Cancer Ther 2021;20:1572–83. 10.1158/1535-7163.MCT-20-0962. 34224362 PMC8419089

